# The importance of multiple Z- plasty- assisted physical therapy in the treatment of Dupuytren’s contracture

**DOI:** 10.1186/s13018-024-05387-3

**Published:** 2025-01-07

**Authors:** Nader Gomaa Elmelegy, Dalia Nader

**Affiliations:** 1 Department of Plastic Surgery, Faculty of Medicine, Tanta University, Tanta, Egypt; 2 Department of Physical Medicine, Rheumatology and Rehabilitation, Faculty of Medicine, Tanta University, Tanta, Egypt

**Keywords:** Dupuytren’s disease, Fasciectomy, Dermofasciectomy

## Abstract

**Background:**

The palmar aponeurosis is extremely adherent to the skin above it. Many of the pre-tendinous coarse fibers enter the dermis at an angle, not just in the palmar creases but also throughout the palm. It’s difficult to distinguish whether Dupuytren’s illness starts in the skin’s dermis or the palmar aponeurosis since the skin adheres so closely to the palmar fascia. In this work, we have investigated the clinical and histological origins of Dupuytren’s disease, as well as its impact on the disease’s management.

**Methods:**

A clinical prospective study was conducted on 47 patients, 42 males and 5 females, who presented with Dupuytren’s contracture in the hands (29 patients were bilateral and 18 one-sided), in the period between April 2012 and September 2020.

**Results:**

Histologically, all our specimens showed chronic inflammatory skin lesions showing hyperkeratotic epidermal covering and dermal infiltration with aggregates of chronic inflammatory cells, mainly lymphocytes and plasma cells, proliferated vascular spaces, and fibrous stroma. Clinical satisfaction was excellent in 67(88.2%) hands, good in six (7.8%)hands, fair in three (4%) hands, and no poor results.

**Conclusions:**

Dupuytren’s disease is a chronic inflammatory skin illness that can penetrate fascia, as we’ve proved histologically and surgically. For a considerable reduction in recurrence, the adhering skin and accompanying cord must be removed.

**Level of evidence:**

IV – therapeutic study.

**Supplementary Information:**

The online version contains supplementary material available at 10.1186/s13018-024-05387-3.

## Introduction

Dupuytren’s disease is a fibro-proliferative condition that affects between 0.6 and 31.6% of the world’s population [1]. Despite its significant morbidity and associated healthcare costs, many aspects of its pathogenesis, classification, and therapy remain debatable [2].

The palmar aponeurosis is extremely adherent to the skin above it. Many of the pre-tendinous coarse fibers enter the dermis at an angle, not just in the palmar creases but also throughout the palm. The numerous little vertical bands superficial to the palmar aponeurosis, which likewise enter the dermis, are not the same as these [3].

It’s difficult to distinguish whether Dupuytren’s illness starts in the skin’s dermis or the palmar aponeurosis since the skin adheres so closely to the palmar fascia.

The ultrastructural study of Dupuytren’s disease revealed that there is no definitive line between the collection of fibroblast-like cells and nearby normal tissue and that the fibroblasts at the hyper-cellular foci appear to merge with the tissue collagen of the aponeurosis. In contrast to desmoid tumors, there was no evidence of purposeful muscle invasion [4].

By comparing the ultrastructure of the fibrous tissue in both disorders, identical myofibroblast cells in both carpal tunnel syndrome and Dupuytren’s disease have been reported. It should also be noted that the presence of myofibroblasts is not exclusive to Dupuytren’s disease [5].

In immune-histological studies by Massimiliano et al. [5], it was discovered that only a few cells express vimentin in the proliferative stage, which is more visible in scar tissue. It has recently been discovered that cells of aggressive fibromatoses display the Ki-1 antigen (CD30), which was previously thought to be restricted to activated lymphocytes and malignancies.

The replicating cells are particularly prevalent in vascular or perivascular regions. Furthermore, the vital uniqueness of fibroblast proliferation begins around confined micro-vessels [6].

Mast cells and fibrotic disorders have recently received a lot of attention, and it’s been suggested that either mast cells or histamine are to blame for the enhanced fibroblast growth and collagen union. Histological examination of Dupuytren’s disease, from another vision, rarely reveals mast cells, which are found either peri-vascular or within the connective tissue cells of the nodules. This could show that neither mast cells nor their products are involved in the disease’s development [7].

The pathophysiology of Dupuytren’s disease can be explained in two ways. From one perspective, Dupuytren’s disease can be classified as an infiltrative disease. The infiltrative disorder is caused by the differentiation of blood-borne cells. This type of mechanism has recently been postulated as a key stage in the creation of nodules. Furthermore, in the case of Dupuytren’s disease, there are no reports in the literature that support such a view [8].

Dupuytren’s disease, on the other hand, is considered a proliferative disease similar to fibro-connective sarcoma’s tissue proliferation. According to some authors, a transitional cell called the fibro-histiocyte is responsible for separating connective tissue cells into distinct types [9].

Because neither the etiology nor the pathogenesis of Dupuytren’s disease has been explained, the assessment remains valid. In this work, we have investigated the clinical and histological origins of Dupuytren’s disease, as well as its impact on the disease’s management.

## Patients & methods

### Materials and methods

A clinical prospective study was conducted on 47 patients, 42 males and 5 females, who presented with Dupuytren’s contracture in the hands (29 patients were bilateral and 18 one-sided). The patients were between the ages of 47 and 69, with a median age of 53.

### Inclusion criteria

This study covered all patients who presented to Tanta University hospital, the plastic surgery department, or a private clinic, with Dupuytren’s contracture in the form of a cord or nodule causing flexion of the metacarpophalangeal and interphalangeal joints of the hand above 30 degrees, in the period between April 2012 and September 2024.

Questions about family history, ectopic areas of involvement, bilateral involvement, and patient age at onset were asked of all patients with suspected Dupuytren’s contracture.

### The limitations of the work

Patients with immune problems, those who had already undergone surgery on the afflicted hand, and those with rheumatoid arthritis or other connective tissue illnesses were all excluded from the study.

Each patient in this study signed a written consent form that contained details about the potential for negative results, consent to clinical photography, and the possibility of their data being published in medical journals.

### Setup

Intravenous antibiotics were given before the start of anesthesia. The patient was placed in a supine position with the aid of a hand table. The limb was exsanguinated with an Esmarch elastic bandage and an inflatable tourniquet. Operative procedures are performed under local intravenous anesthesia with regional peripheral nerve blockade. The upper extremity is draped after complete sterilization using povidone-iodine.

### Skin incisions

**Fig. 1:**
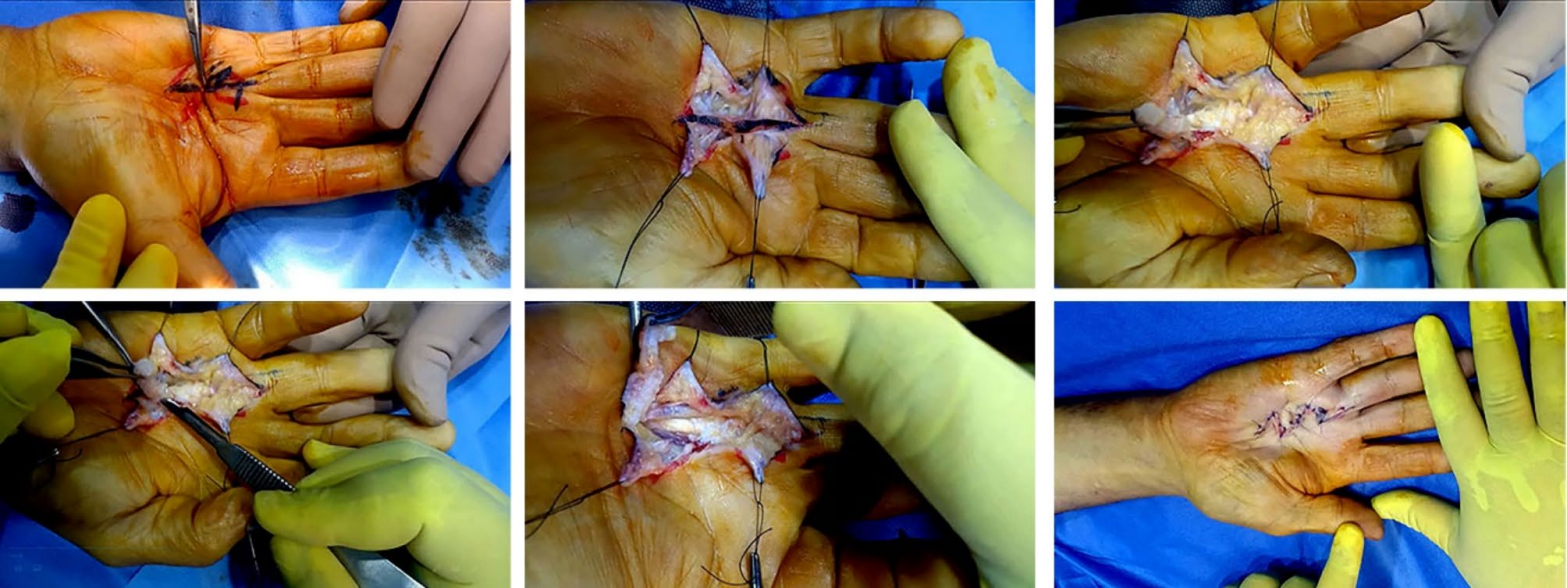
Upper left: Two to three millimeters of the adherent skin were marked along the whole length of the cord, as well as two Z plates on the outside and inner skin. Upper middle: Two parallel longitudinal skin incisions along the previously marked area were made. The outer and inner flaps of healthy tissue over the palm and digits are dissected. Upper right: the cord was resected on-mass from distal to proximal with two to three mm of adherent skin revealing subcutaneous fat beneath the cord. Lower left: Sharp dissection was also performed at the proximal end of the chord. Lower middle: Complete resection of diseased tissue and cords, revealing subcutaneous fat beneath the cord lower right. Finally, the wound was closed in a zigzag pattern

Two to three mm of the adherent skin were marked along the whole length of the cord, as well as two Z plates on the outside and inner skin Fig. 1a. Two parallel longitudinal skin incisions, two to three mm apart, along the previously marked area, were made, commencing just proximal to the cord and finishing just distal to the cord or nodule Fig. 1b. The outer and inner flaps of healthy tissue over the palm and digits are dissected away to allow good exposure of the structures in-between Fig. 1b & c.

### Dissection

The preoperative evaluation of the hand informs the surgeon about which pathologic cords to expect during the surgical exposure, thereby reducing the chance of neurovascular injury. The pre-tendinous, central, natatory, and other cords are commonly removed. Because there was a layer of fat underneath the cord in the area from the natatory ligament to the terminal end, the cord was resected from distal to proximal en-mass with the two to three mm of adherent skin Fig. 1d. This simplified the dissection, offered good exposure, and helped safeguard all the important structures underneath the cord. Sharp dissection was also performed at the proximal end of the chord since the skin has tightly adhered to the palmer fascia (Fig. 1e).

Resection of diseased tissue and cords reliably corrects MCP and PIP joint contractures to a significant degree. However, to achieve the anticipated degree of improvement and allow easy skin closure, multiple z-plasty over the outer and inner healthy skin flaps were performed. At the end of the procedure, the tourniquet was released and special care was paid to achieving hemostasis. Before closure, the vascularity of all digits must be checked. Vasospasm can be treated using warm sponges just after the tourniquet is released.

Interrupted 4 − 0 Prolene sutures were used to close the wound Fig. 1f. Upon closure, one layer of tulle grass is put to the wound once it has been closed, followed by a polyvinyl sponge or dry gauze. A volar plaster slab is placed over the soft dressing and maintained in place with sustained pressure until the plaster splint hardens and the MCP, PIP, and DIP joints are in the proper extension position. Seven to ten days after surgery, the stitches were removed. Histological analysis of the resected cord in its entirety, including the adhering skin, was performed.

## Results

This study comprised 47 patients with Dupuytren’s contracture, which was bilateral in 29(61.7%) patients and one-sided in 18 (38.2%) others. In total. 76 hands were affected by the condition, the left hand was affected in 47(61.8%) cases, whereas the right hand was affected in 29 (38.2%) cases. In 31 (40.8%) hands, the ring finger was afflicted alone, the little finger is affected in 8 (10.5%), the ring and little fingers are affected in 27(35.5%), and the ring and middle fingers are affected in ten (13.2%).

Before the patient was presented to us, the condition had lasted three years in 28(59.6%) people, 4 years in nine (19.1%) patients, five years in six (12.8%) patients, and six years in four (8.5%) patients.

Forty-two (89.4%) of the patients were men, whereas five (10.6%) were women. Eleven (23.4%) of the patients were diabetic, 17 (36.1%) had a hard time with their hands, and seven (14.9%) were habitual smokers. Nine (19.1%) of the patients had a positive family history of Dupuytren’s disease in a close relative. There was no disease on the plantar surface of the patients’ feet.

These patients were followed for an average of 4.2 years (range: 1–7 years). After this treatment, 67 (88.2%) of the 76 hands had a complete range of motion in both flexion and extension, while 9 (11.8%) had 5–10 degrees of proximal interphalangeal joint flexion contracture. One of these patients had a finger that was fixed (a 5-degree correction) at the time of surgery, whereas the other eight had fingers that were only partially cured (residual contracture > 5 degrees).

In our examination, there was no loss of flexion or vascular or nerve injury associated with this condition. There were two (2.6%) occurrences of hematoma that formed shortly after surgery but responded promptly to repeated suction, effective anti-inflammatory, and antibacterial treatment. In two (2.6%) individuals, the wound became infected, but with medicines and regular dressings with povidone-iodine, the wound healed without complications. Three of the repaired hands developed wound dehiscence, which healed without incident with daily dressing using povidone-iodine.

Clinical satisfaction testing was carried out. The outcomes were rated as excellent, good, fair, and bad on a scale of one to four. Three plastic surgeons who were not involved in the study assessed the results by comparing preoperative, follow-up, and late photos. When the score was (4), the results were judged excellent, good when it was (3), fair when it was (2), and poor when it was (1). Clinical satisfaction was excellent in 67(88.2%) hands, good in six (7.8%)hands, and fair in three (4%) hands.

Patient satisfaction with their restored hand function, lifestyle, remarks from family, and overall contentment was assessed by using PROMs. Results were reported according to a scale from 0 to 4 as follows: 0–1 indicates poor performance, 2 indicates fair performance, 3 indicates good performance, and 4 indicates excellent performance. Patient satisfaction was excellent in 63 (82.9%) hands, good in seven (9.2%) hands, and fair in six (7.9%) hands.

**Fig. 2:**
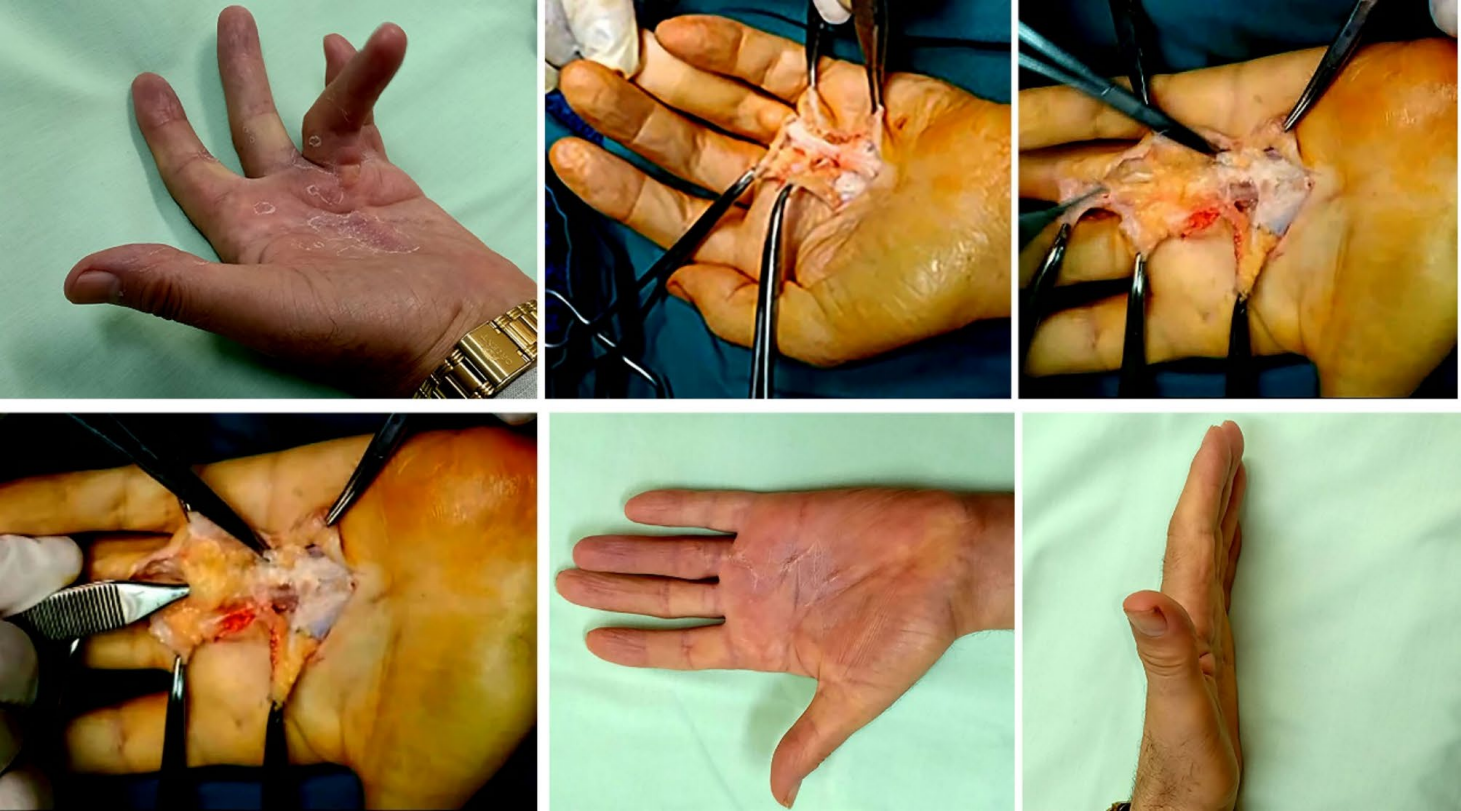
Upper left. Preoperative photos of a 62-year-old right-handed man with Dupuytren’s disease affecting the MPJ and PIPJ in the left ring finger. Upper middle. The cord is entirely exposed once the adherent skin is removed, with no subcutaneous fat in between. Upper right & lower left. The subcutaneous fatty layer beneath the cord is seen. Lower middle & left. Three years after surgery, showing complete repair of the deformity

**Fig. 3:**
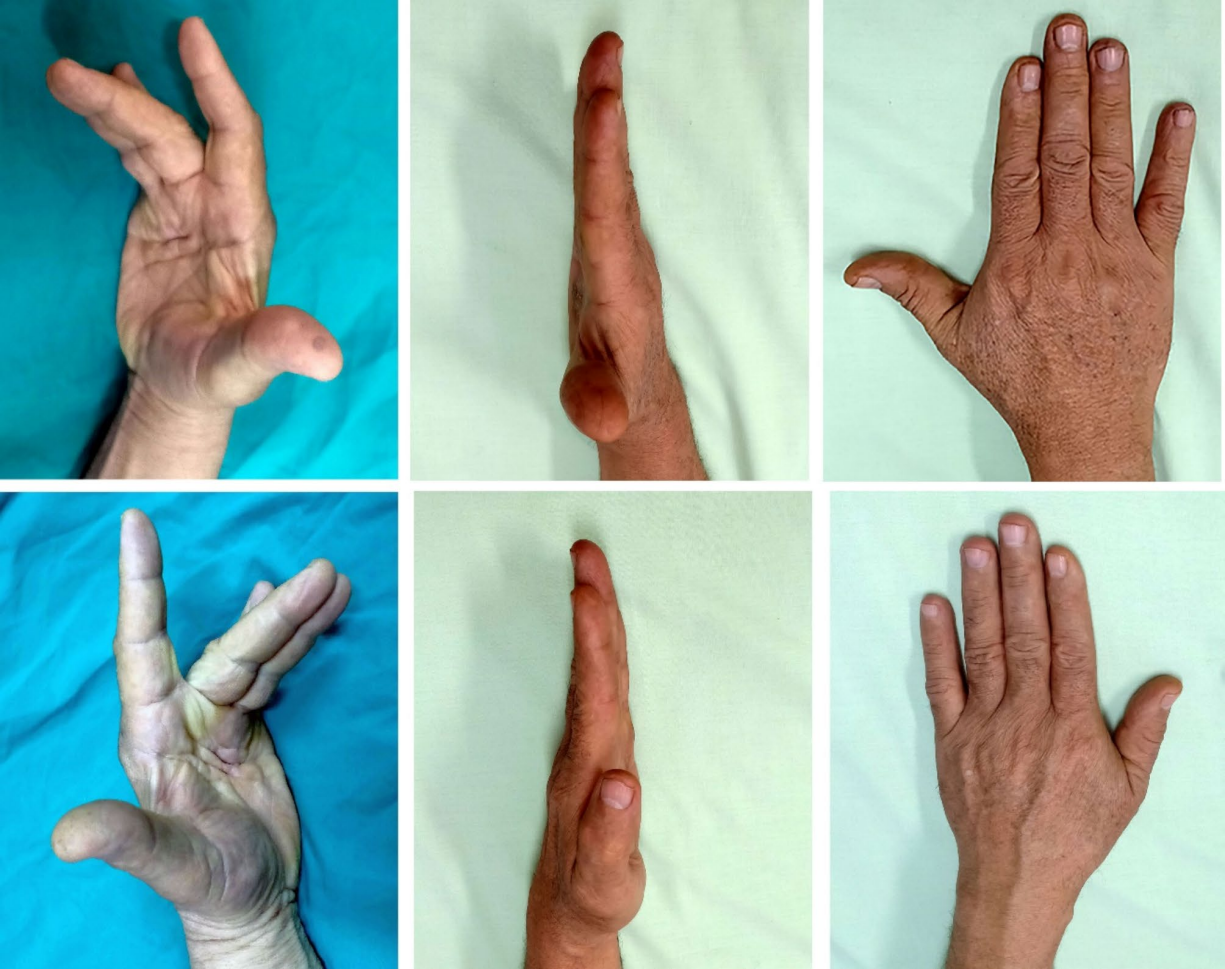
Upper left. and lower left. Preoperative images show a 59-year-old man with Dupuytren’s disease in both the right and left ring and middle fingers, as well as the MPJ and PIPJ. Upper middle & right, lower middle & right. One year after surgery, the deformity is completely corrected

**Fig. 4:**
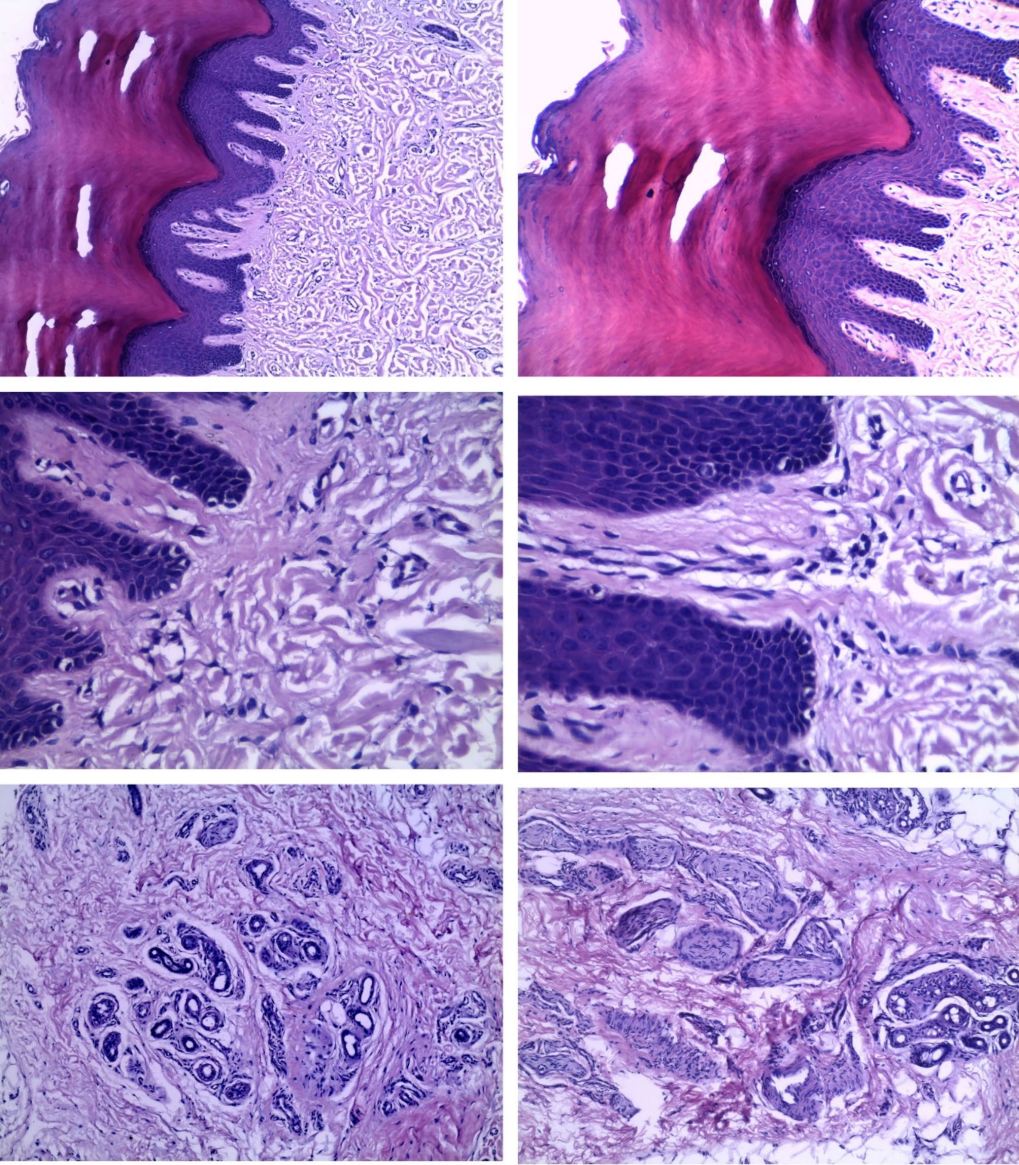
Upper left& upper right: Histologically, all our specimens showed Inflammatory lesions with hyperkeratotic epidermal covering middle right & middle left, dermal infiltration with aggregation of chronic inflammatory cells, primarily lymphocytes and plasma cells, lower right & lower left, proliferating vascular spaces, and fibrous stroma

Histologically, all our specimens showed inflammatory lesions showing hyperkeratotic epidermal covering and dermal infiltration with aggregates of chronic inflammatory cells, mainly lymphocytes and plasma cells, proliferated vascular spaces, and fibrous stroma. No granuloma and no malignant changes could be detected. Table 1; Figs. 2 and 3, and 4 (Supplementary material 1: Video).

**Table 1:** Descriptive analytic data of different variability in age, sex, and duration of the disease before it was presented to us, number of affected hands, affected hand location, affected fingers, clinical satisfaction, patient satisfaction, and complications encountered

Variable	Total number of patients (47)	Percentage
Age		
40–50 y	7	14.9%
50–60 y	31	66%
Above 60 y	9	19.1%
Sex		
Male	42	89.4%
female	5	10.6%
Duration of the disease before presented to us:		
Three years	28	59.6%
Four years	9	19.1%
Five years	6	12.8%
Six years	4	8.5%
Number of affected hands		
Unilateral	18	38.3%
bilateral	29	61.7%
Site of the affected hands		
Left hand	47	61.8%
Right hand	29	38.2%
Fingers affected		
Ring	31 fingers	40.8%
Little	8 fingers	10.5%
Ring and little	27 fingers	35.5&
Ring and middle	10 fingers	13.2%
Clinical assessment		
Excellent	67 hands	88.2%
Good	6 hands	7.8%
Fair	3 hands	4%
Bad	0	0%
Patient satisfaction:		
Excellent	63 hands	82.9%
Good	7 hands	9.2%
Fair	6 hands	7.9%
Bad	0	0%
Complications encountered:		
Hematoma	2	2.6%
Infection	2	2.6%
Wound disruption	3	4%
Under correction	9	11.8%

## Discussion

The significance of the palmar skin in Dupuytren’s disease formation, propagation, surgical therapy, and recurrence risk, remains uncertain. Dupuytren’s disease patients can be treated with many different therapies. Only mild disorders of the disease can be observed. Collagenase injections intra-lesional, percutaneous needle fasciotomy, or selective aponeurectomy are all options, although progression or recurrence occurs in up to 85% of instances [10, 11].

Although up to 100% of patients have a recurrence, limited fasciectomy [12] is the most common treatment for moderate to severe conditions. Hueston [13] proposed skin replacement as a treatment for recurrent Dupuytren’s disease in the 1960s. Gonzalez [14] came up with this theory, emphasizing the need to remove all pre-axial tissue (skin, fat, and fibrous tissue) and replace the defect with a full-thickness skin graft (FTSG) [15, 16].

The same group later discovered fibromatosis in the skin of individuals with recurring Dupuytren’s disease, leading to the recommendation of dermo-fasciectomy as the best surgical choice [17]. Since then, studies have shown that dermo-fasciectomy and full-thickness skin grafts can reduce the incidence of recurrence by up to 33% [18, 19].

In our research, we removed 2–3 mm of adhering skin along with the entire cord. At the commencement of the cord, we also removed the adhering fascia. We did not remove any fascia beyond the natatory ligament since we found that there was a good subcutaneous layer under the cord and covering the fascia of the digits. In none of our situations has there been a recurrence. This is due to the fact that we assumed the disease-causing tissue was the skin rather than the fascia when we removed it.

There is limited literature comparing the clinical and histological features of Dupuytren’s disease in the skin [20] and there are no reports on microscopic examination of clinically uninvolved skin [14]. Wade et al. [21] discovered cutaneous fibromatosis in 61% of histopathological cases. In addition, 22 of the 44 patients with dermal involvement had no clinical symptoms of skin involvement and were treated with fasciectomy. This finding supports our hypothesis that Dupuytren’s illness begins in the dermis before progressing to fascia involvement and manifesting clinically.

All our specimens showed inflammatory lesions with hyperkeratotic epidermal covering and dermal infiltration with aggregation of chronic inflammatory cells, primarily lymphocytes, and plasma cells, proliferating vascular spaces, and fibrous stroma. There were no granulomas or malignant alterations found. This further supports our theory that this is a chronic inflammatory skin condition that begins in the deep dermis at stress points. Because the palmar fascia is very adherent to the dermis in the palm, it appears to originate there. However, as we have demonstrated clinically, the cord is separated from the fascia by a well-defined subcutaneous layer distally.

In limited/small skin biopsies, histologically distinguishing Dupuytren’s fibromatosis from hypertrophic scarring is not always achievable. Because the tissue in both circumstances has enhanced cellularity and fibroblastic activity, the morphological traits are identical. Hypertrophic scars, on the other hand, usually have thick collagen bundles and do not create “burned out” fibrotic nodules, which are common in late-stage Dupuytren’s disease [22], As a result, the general morphology plays a big role in the diagnosis. On the other hand, one could argue that distinguishing between dermal fibromatosis and excessive scarring is unnecessary because the most important task is to remove all fibrotic tissue, which is also the conclusion of our study, which found that this disease should be treated as strongly fibrotic scar tissue that passes over joints, which is why we have performed multiple z-plasty to prevent a recurrence.

Gonzalez [14] demonstrated that dermo-fasciectomy substantially excised skin, fat, fascia, aponeurosis, scar, and diseased tissue, resulting in a significant improvement in range of motion. However, they are unable to fully explain why their dermo-fasciectomy patients have straighter digits and advise that this is an issue that should be researched further. However, we can attribute this improvement to the fact that the disease began on the skin, which was completely removed, reducing the risk of recurrence.

Anecdotally, some surgeons discourage the use of dermo-fasciectomy due to the alleged risk of graft loss, perceived surgical complexity, and longer rehabilitation. To date, no studies have demonstrated a statistically or clinically significant risk of graft loss. According to anecdotal evidence, these patients take longer to resume normal daily activities, which should be balanced against a potentially lower rate of recurrence and revision surgery [23, 24]. We performed restricted surgery in our study, removing the chord and the adhering skin above it, and had great results with no recurrence. We do not have a skin graft; therefore, we’ve avoided all of the difficulties that come with them. Our patients began working one to two weeks after the stitches were removed.

The use of hand therapy in the treatment of Dupuytren’s disease has been recommended in numerous studies [25]. Hand therapy as a prophylactic treatment for Dupuytren’s disease has insufficient evidence. Hand physical therapy was individualized to each patient’s needs after corrective treatment and includes orthotics, exercise, edema control, and pain or scar management. Because we had limited surgery and did not touch the fibrous flexor sheath in our study, we did not refer our patients to the physiotherapy department.

## Conclusions

We have demonstrated both histologically and surgically that Dupuytren’s disease is a persistent inflammatory skin condition that could pierce fascia. Removal of the adherent skin and associated cord is necessary for a significant decrease in recurrence. Because the resulting scars spread over the joints, Z-plasty is necessary to prevent scar recurrence.

## Electronic supplementary material

Below is the link to the electronic supplementary material.

Supplementary Material 1: Video

## Data Availability

No datasets were generated or analysed during the current study.
